# Drug Repositioning Screen on a New Primary Cell Line Identifies Potent Therapeutics for Glioblastoma

**DOI:** 10.3389/fnins.2020.578316

**Published:** 2020-12-17

**Authors:** Filiz Senbabaoglu, Ali Cenk Aksu, Ahmet Cingoz, Fidan Seker-Polat, Esra Borklu-Yucel, İhsan Solaroglu, Tugba Bagci-Onder

**Affiliations:** ^1^Brain Cancer Research and Therapy Laboratory, Koç University School of Medicine, Istanbul, Turkey; ^2^Koç University Research Center for Translational Medicine, Istanbul, Turkey; ^3^Medical Genetics Department and Diagnostic Center for Genetic Diseases, Koç University Hospital, Istanbul, Turkey; ^4^Department of Neurosurgery, Koç University School of Medicine, Istanbul, Turkey; ^5^Department of Basic Sciences, Loma Linda University, Loma Linda, CA, United States

**Keywords:** glioblastoma, primary cell line, drug repurposing, Topotecan, Temozolomide

## Abstract

Glioblastoma is a malignant brain cancer with limited treatment options and high mortality rate. While established glioblastoma cell line models provide valuable information, they ultimately lose most primary characteristics of tumors under long-term serum culture conditions. Therefore, established cell lines do not necessarily recapitulate genetic and morphological characteristics of real tumors. In this study, in line with the growing interest in using primary cell line models derived from patient tissue, we generated a primary glioblastoma cell line, KUGBM8 and characterized its genetic alterations, long term growth ability, tumor formation capacity and its response to Temozolomide, the front-line chemotherapy utilized clinically. In addition, we performed a drug repurposing screen on the KUGBM8 cell line to identify FDA-approved agents that can be incorporated into glioblastoma treatment regimen and identified Topotecan as a lead drug among 1,200 drugs. We showed Topotecan can induce cell death in KUGBM8 and other primary cell lines and cooperate with Temozolomide in low dosage combinations. Together, our study provides a new primary cell line model that can be suitable for both *in vitro* and *in vivo* studies and suggests that Topotecan can offer promise as a therapeutic approach for glioblastoma.

## Introduction

Glioblastoma (grade IV astrocytoma) is the most common of all malignant brain and CNS tumors (Ohgaki and Kleihues, 2005). Relative survival estimates for glioblastoma patients are quite low; 5.1% of patients survive 5 years post diagnosis. The hallmarks of glioblastoma can be defined as uncontrolled cell proliferation, diffuse infiltration, tendency for necrosis, angiogenesis, resistance to apoptosis, and genomic instability. The aggressive nature and heterogeneity of glioblastoma make treatment very difficult: Extensive tumor infiltration into the surrounding healthy brain tissue, tumor mass formed by distinct cell types and resistance to available therapy options are major challenges for successful treatment (Cancer et al., 2008; Ostrom et al., 2015; Mansouri et al., 2017).

Current therapies include surgery, radiation therapy, and administration of Temozolomide (TMZ). TMZ is usually administered at the beginning of treatment along with radiotherapy for 6 weeks, followed by six cycles of TMZ alone for 5 days every 28 days. With this standard regimen, the median survival reaches 14.6 months compared to 12 months observed for patients treated with radiotherapy alone (Stupp et al., 2009). This aggressive trimodal regimen is still faint against glioblastoma, recurrence occurs within months in 90% of patients (Stummer et al., 2005; Stupp et al., 2009). Upon recurrence, many patients undergo further surgical resection if possible; and therapy options include re-challenging with alkylating agents such as TMZ, platinum-based drugs, or VEGF inhibitors (Pavon et al., 2014; Messaoudi et al., 2015). Yet, none of these options can currently cure glioblastoma.

For years, glioblastoma cell lines such as U87MG, U251, and T98G, have been studied extensively, providing valuable information about this cancer type. Cell line-based models are reproducible with reliable growth rates for unlimited number of divisions (Huszthy et al., 2012). However, cell line-based models also have shortcomings: First, the serum-containing medium modifies both their genomes and transcriptomes. Prolonged cell culture changes the genetic, epigenetic, and morphological characteristics of cancers. For example, most glioblastoma lines lack epidermal growth factor receptor (EGFR) amplification, while 40% of the primary tumor specimens contain this mutation. In addition, prolonged cell culture leads to progressive hypermethylation of the MGMT promoter, approximately 80% of glioblastoma cell lines are MGMT hypermethylated, as compared to a 40% frequency of promoter hypermethylation in the initial clinical diagnosis (Carlson et al., 2011). Second, upon orthotopic implantation to the brain, most cell lines fail to develop the defining morphological features of glioblastoma, such as diffuse infiltration into surrounding healthy tissue and microvascular proliferation (Lee et al., 2006; Xie et al., 2015). Third, including serum in the cell culture media reduces tumor-propagating and stem cell-like feature of tumor cells due to cellular differentiation (Singh et al., 2003; Seidel et al., 2014). Thus, serum-based 2D monolayer cultures are not the best option to investigate therapeutic effects of a new finding in glioblastoma.

Glioblastoma cell culture techniques have been improved in the last years by culturing glioblastoma cells in serum-free medium that was originally established for neural stem cells (NSCs) (Singh et al., 2003; Galli et al., 2004). Neurobasal media, a DMEM derivative with reduced osmolarity and lower glutamine concentrations, are supplemented with biotin, insulin, transferrin, FGF and EGF (Brewer et al., 1993; Ledur et al., 2017). In this defined medium, NSCs and glioblastoma cancer stem cell like cells maintain their self-renewal ability and display no differentiation (Singh et al., 2003; Quiñones-Hinojosa et al., 2007). Additionally, in this serum-free NSC media, glioblastoma cells grow as spheres, resembling neurospheres, and they can keep a semi-3D environment in which cells deposit ECM to create their own unique microenvironment (10). Orthotopic transplantation of spheroid glioblastoma cells generates secondary tumors, which retain the features of the primary tumors that the cells are originally derived from. Moreover, direct orthotopic transplantation of patient derived glioblastoma cells show that these xenograft models represent the histopathology, genetic and phenotypic properties of the corresponding patient’s primary tumor (Xie et al., 2015). Thus, while investigating glioblastoma, it is essential to work with primary cell cultures and/or xenografts if possible.

Drug repurposing or repositioning is the application of known drugs to other uses, which eliminates years required to develop a new drug and provides a marked economic benefit (Cha et al., 2018). In addition, a repurposed drug already comes with extensive preclinical and clinical knowledge, which can reduce cytotoxicity issues for the newly desired application. Drug repurposing has provided many potential candidates for various diseases in the past. For example, a screen in 1994 revealed Gefitinib (AstraZeneca) and Erlotinib (Roche) as specific inhibitors of the EGFR, which were approved by Food and Drug Administration (FDA) in 2003, and are currently used in the treatment of many cancers (Fry et al., 1994).

In this study, we first generated a new primary glioblastoma cell line, called KUGBM8, and characterized its *in vitro* and *in vivo* growth properties as well as its response to TMZ. Providing a new cell line model, we conducted a high throughput screen with a 1,200 FDA-approved drug library as part of a drug repurposing approach. We identified Topotecan, a Topoisomerase inhibitor, as the lead agent that inhibited the growth of KUGBM8 cells, suggesting its clinical potential for glioblastoma therapy.

## Materials and Methods

### Patient Information

**FIGURE 1:**
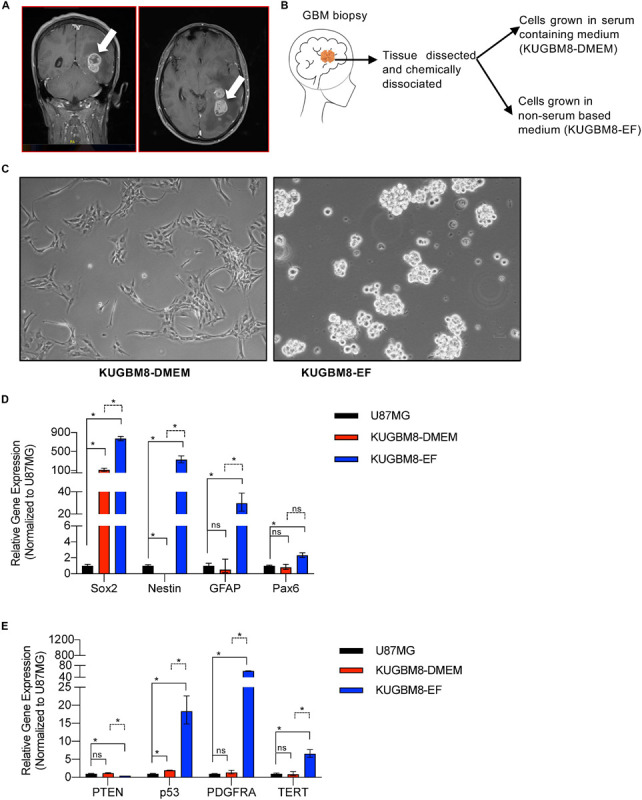
Establishment of primary KUGBM8 cell lines. **(A)** MRI brain gadolinium-enhanced T1 weighted image (coronal and sagittal view, respectively) showing hyperintense lesion (arrows) in the left parieto-occipital lobe. **(B)** Schematics of cell line establishment from the tumor. **(C)** Representative photos of KUGBM8-DMEM and KUGBM8-EF cell lines taken by 10X light microscopy. **(D)** Gene expression levels of neural stem cell marker genes detected by qRT-PCR. Values are normalized to the housekeeping gene GAPDH. **(E)** Gene expression levels of cancer related genes detected by qRT-PCR. Values are normalized to the housekeeping gene GAPDH. * denotes *p* < 0.05, ns denotes *p* > 0.05, *t*-test.

A patient within the range of 40–45 year-old was presented with a 2 months history of headache, right hemiparesis and impairment of vision. Magnetic resonance imaging (Figure 1A) studies revealed a hyperintense lobulated tumor in the left parieto-occipital lobe. The patient underwent a left parieto-occipital craniotomy. The tumor was removed gross totally. Histopathology of the tumor confirmed the diagnosis of glioblastoma. The postoperative course was uneventful, and patient was discharged from the hospital on the fifth postoperative day.

### Establishing Primary Glioblastoma Cell Line KUGBM8

Protocol of primary cell line formation was adapted from Xie et al. (2015) and conducted according to the guidelines and approval of the Koç University Institutional Review Board (2014.079.IRB2.022). Surgically removed tumor specimens were placed in DMEM containing 2.5% Fetal Bovine Serum (FBS) and 1% Pen-strep prior to transfer on ice. Fresh tumor samples were washed with 1X PBS containing 1% Pen-strep several times and minced with a scalpel.

The dissected tissue was incubated in a mixture of 1:1 Accutase (Stem Cell Technologies) and Trypsin (Gibco) for 10 min at 37°C for chemical dissociation. The dissociated cells were washed twice with DMEM/F12, followed by centrifugation at 600 rpm for 8 min. Tumor sample was then divided into two parts, one part was transferred into wells of a 6-well plate (depending on the size of the tumor specimen) and supplemented with DMEM containing 10% FBS and 1% Pen-strep. The attachment of the cells from the dissected tissue was observed and medium was changed 2–3 times a week. These cells were named KUGBM8-DMEM. Second part of the dissociated tissue was plated to 25 cm^3^ flasks with EF medium [Neurobasal medium (Gibco, Cat. No. 21103-049), L-Glutamine (Gibco), B27 and N2 supplements (Gibco), Pen-Strep (0.5%, Gibco), Heparin (Stem Cell Technologies), FGF (20 ng/ml, Gibco, PHG0266), and EGF (20 ng/ml, Peprotech, AF-100-15)]. After sphere formation (5–7 days), spheres in suspension were centrifuged and divided into single cells with accutase. Resuspended cells were placed in a new flask containing fresh media. These cells were named KUGBM8-EF. Cells were kept in 37°C humidified incubator with 5% CO_2_. Cells were routinely checked for mycoplasma contamination (Lonza Mycoplasma Detection Kit).

### Cell Line Oncopanel Testing Capture

Patient derived cell lines KUGBM8-DMEM was KUGBM8-EF was studied by Oncopanel testing capture (Illumina’s TruSight Cancer Kit, Illumina), by screening 94 genes and 284 SNPs as described before (Börklü-Yücel et al., 2020). Briefly, sequencing was performed using MiSeq sequencer (Illumina) to produce 2 × 150 bp reads. Raw reads were mapped against human reference genome hg19 using BWA-MEM algorithm, de-duplicated using Picard and variant calling was executed using GATK best practices pipeline. Quality filtered variants with a minimum of 20X coverage and no more than 10% Mapping Quality Zero (MAPQ0), were annotated using ANNOVAR with avSNP release of 142, 1,000 genomes release of 2014 along with NIH-NHLBI 6500 exome database version 2. MAF filter (< 0.01) was set based on ExAC, NIH 6500, gnomAD, and 1,000 genomes data. Pathogenicity evaluation was performed based on the inheritance mode, database entries (HGMD, ClinVar, CentoMD), *in silico* prediction tools (SIFT, Polyphen2, MutationTaster) and ACMG recommendations. All intronic variants located outside the boundaries of 10 bp from the exons and synonymous exonic ones were filtered out. Putative splicing variants were analyzed using Human Splicing Finder (HSF). Sequencing data is deposited to SRA database (PRJNA644513).

### Cell Culture

Human glioblastoma cells U87MG was supplied and authenticated by American Tissue Type Culture Collection (ATCC) and cultured in DMEM (Gibco) with 10% Fetal Bovine Serum (Gibco) and 1% Pen-Strep (Gibco). GBM4 and GBM8 patient derived glioblastoma cells were kind gifts from Dr. Hiroaki Wakimoto (Massachusetts General Hospital, Boston, MA) and cultured in Neurobasal medium (Gibco, Cat. No. 21103-049), L-Glutamine (Gibco), B27 and N2 supplements (Gibco), Pen-Strep (0.5%, Gibco), Heparin (Stem Cell Technologies), FGF (20 ng/ml, Gibco, PHG0266), and EGF (20 ng/ml, Peprotech, AF-100-15). KUGBM8-EF and KUGBM8-DMEM cells were established and cultured as described above.

### Quantitative Real Time PCR (qRT-PCR)

U87MG, KUGBM8-DMEM, and KUGBM8-EF cells were collected, and total RNA was isolated using Macherey-Nagel RNA Nucleospin isolation kit (MN GbmH & Co.) according to manufacturer’s protocol. 1 μg of RNA was used to establish cDNA with M-MLV Reverse Transcriptase (Invitrogen). Quantitative real time PCR was performed with PikoReal 96 Real Time PCR system (Thermo Fisher Scientific) by using LightCycler 480 SYBR Green I Master (Roche). The primers used in these experiments are listed in Supplementary Table 1. Relative gene expression levels were detected by normalization to GAPDH expression.

### Testing Tumor Forming Capacities and Temozolomide Response *in vivo*

SCID mice cared in appropriate conditions of Koç University Animal Facility were used and the institution boards of Koç University (HADYEK#2014-22) approved all protocols. Firefly Luciferase (Fluc) and mCherry expressing stable KUGBM8-DMEM and KUGBM8-EF cells were generated by viral transduction as described (Bagci-Onder et al., 2012). Initially, mCherry expression was examined under fluorescence microscopy and their Fluc activity was validated by utilizing *in vitro* luminescence assay. Increasing number of Fluc-mCherry expressing cells (0–50.000/well) were seeded on 96-well black-bottom plates and luciferase activity was detected by adding 1,000 μg/ml D-Luciferin. Bioluminescence measurements were taken every week.

For tumor implantation, 4 mice for each condition were used. A total of 120,000 cells per mouse were injected in 7 μl PBS stereotactically into the brain (From bregma, AP: −2 mm, ML: 1.5 mm and from dura V: 2 mm). After injection, tumor growth was visualized using IVIS Lumina III Bioluminescence Imager. Accordingly, Fluc activity of tumors was measured by injecting mice with 150 μg/g body weight of D-Luciferin intraperitoneally. After repeated measurements of tumor growth for 75 days, mice were sacrificed, and brains were recovered with perfusion. Quantification of tumor progression was performed with GraphPad PRISM software. For histological analyses, 10 micron thick cryo-sections from tumors were stained with hematoxylin and eosin and imaged with Leica M205 FA Stereo microscope (Leica Microsystems).

For Temozolomide *in vivo* treatment, after tumor formation as gaged by Fluc imaging, mice were treated with 2 mg/kg DMSO or Temozolomide for 5 consecutive days. Growth was assessed by Fluc activity as described above. Statistical measurements were performed using ANOVA.

### Drug Screening and Cell Viability Measurement

The drug library composed of 1,200 FDA (Food and Drug Administration of United States) approved drugs was purchased from Prestwick Chemical (France) as described (Senbabaoglu et al., 2016). Individual drugs, namely Astemizole, Camptothecine (S, +) Quinacrine dihydrochloride dihydrate, Mitoxantrone dihydrochloride, Doxorubicin, and Topotecan were supplied from Prestwick Chemicals.

For the high throughput drug screen, 384-well plates (Corning) were employed. Accordingly, KUGBM8-EF cells were automatically seeded to 384-well plates as 1,500 cells/40 μl/well via Multidrop^TM^ Combi Reagent Dispenser (Thermo Fisher Scientific). Next day, 50 μM drug plates were freshly prepared within EF medium and added to 384 well plates with an automated pipettor (Matrix^TM^ Multichannel Electronic Pipettes, Thermo Fisher Scientific) for a final concentration of 5 μM. Each treatment was performed in quadruplicates. Cell viability was determined 3 days later. Changes in cell viability were measured by normalization to untreated controls. Statistical measurements were performed using *t*-test and IC_50_ of Temozolomide was calculated by Graphpad Prism with non-linear regression analysis.

For the chosen hits, cell viability was further investigated on black 96-well plates (Corning) with different dosages. Cell viability was detected after 3 days after treatment by CTG.

### Western Blot Experiments

Western blots were performed as described previously (Senbabaoglu et al., 2016). Cells were treated with Topotecan for 24 h to check PARP (Cell Signaling/9,542) cleavage. α-Tubulin (Abcam/ab15246) was used as a loading control.

## Results

### Establishment of Patient-Derived Primary Glioblastoma Cell Lines From Fresh Tumor Tissues

KUGBM8-DMEM and KUGBM8-EF cell lines were derived from a glioblastoma patient. MRI brain gadolinium-enhanced T1-weighted image (coronal and sagittal view, respectively) showed hyperintense lesion (arrows) in the left parieto-occipital lobe (Figure 1A). The excised tumor tissue was freshly dissociated and divided into two parts to establish two different cell lines, one that grows under serum-supplied culture conditions (KUGBM8-DMEM) and one growing in an anchorage-independent manner in medium devoid of serum and enriched with neurosphere supplements (KUGBM8-EF) (Figure 1B). The tumor tissue pathological analysis revealed that MGMT promoter was hypermethylated, EGFR was amplified, and PTEN was deleted in the original tumor (Table 1). After cell lines were established, KUGBM8-DMEM cells exhibited typical characteristics of two-dimensional cancer cell line cultures, where cells flattened in a monolayer on the bottom of the culture vessel. On the contrary, KUGBM8-EF cells displayed growth as three-dimensional spheroids (Figure 1C).

**TABLE 1:** Pathology results of the surgically excised tumor tissue.

**Marker**	**Result**
p16	Positive (score 4)
EGFR	Positive (score 3)
Ki-67	Positive (%52)
IDH1	Negative
PTEN	Negative (score 32)
MGMT	Positive (methylated %80–100)
OLIG2	Negative
GFAP	Positive
NeuN	Negative
p53	Negative

To examine the genetic alterations in these established lines, we performed onco-profiling of KUGBM8-DMEM and KUGBM8-EF cells in early passages, and genes were filtered by minor allele frequency (MAF). *MUTYH, PTEN, RB1, TP53*, and *TSC1* (Table 2) genes were found to be commonly mutated in these two cell lines, supporting the proposed role of *TP53*, *PTEN*, and *RB1* in the pathogenesis of human glioblastomas (Chow et al., 2011). Defects in *TSC1*, hamartin, has been proven to induce mTORC1 activity, which in turn triggers glioma development when combined with other oncogenic signals (Yamada et al., 2014), such as variations in the known tumor suppressor genes *PTEN, TP53*, and *RB1*. Biallellic germline mutations of *MUTYH* have primarily been related to colorectal adenomas, but a recent report states that monoallelic germline *MUTYH* mutations may induce an increase in the risk of malignant brain tumors (Kline et al., 2016).

**TABLE 2:** Genetic alterations observed in KUGBM8-EF cells.

**Gene**	**Transcript number**	**Nucleotide change**	**Aminoacid change**	**Zygosity**	**MAF**	**Predicted effect**	**References**
MUTYH	NM_001128425	c.C799T	p.Q267X	Het	0.0002	Pathogenic	PubMed: 17703316
PTEN	NM_000314	c.1026+1G>A	?	Het	−	Pathogenic	PubMed: 15372512
RB1	NM_000321	c.C1654T	p.R552X	Het	−	Pathogenic	PubMed: 7704558
TP53	NM_000546	c.G404T	p.C135F	Het	−	Likely pathogenic	−
TSC1	NM_000368	c.C1798T	p.Q600X	Het	−	Likely pathogenic	−
TSC1	NM_000368	c.G1219A	p.V407M	Het	0.0002	VUS	−

It is widely known that cell culture conditions can alter the genetics and metabolism of cancer cells (Ackermann and Tardito, 2019). To minimize such alterations and resemble the features of patient tumors as much as possible, our newly generated primary cell lines were only used up to passage 10 during the experiments. We also tested how these cells compare to the widely used established cell lines, such as U87MG, for their expression of several genes from the NSC lineage. NSC markers *SOX2*, *NESTIN*, PAX6 (Lathia et al., 2015; Zhang and Jiao, 2015) as well as the well-established astrocyte marker *GFAP* were expressed significantly higher in KUGBM8-EF cells than in KUGBM8-DMEM and U87MG cells (Figure 1D). Next, we checked the gene expression of some cancer related genes (*PTEN, P53, PDGFRA*, and *TERT*) (Figure 1E) and observed significant fold change differences among KUGBM8-DMEM and KUGBM8-EF. For example, high expression of *PDGFR* and *TERT* as well as low expression of *PTEN* was evident in KUGBM8-EF cells compared to KUGBM8-DMEM or U87MG cells.

### Patient Derived Primary Glioblastoma Cell Lines Are Capable of Forming Tumors *in vivo*

**FIGURE 2:**
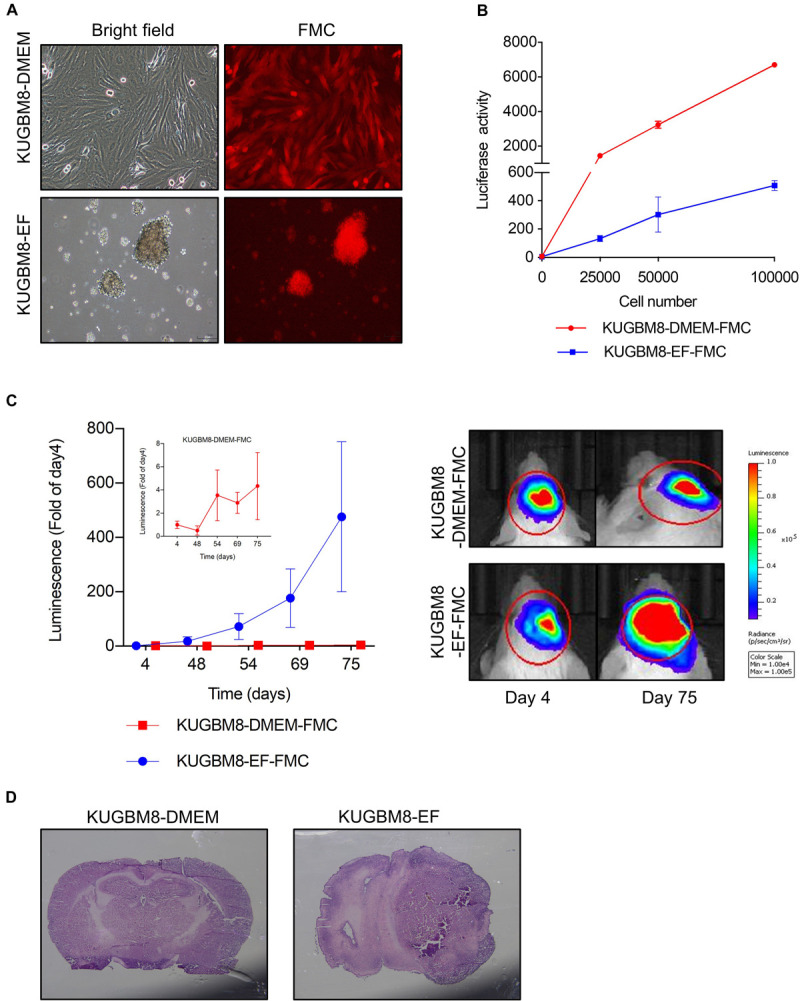
Characterization of KUGBM8 cell lines *in vivo.* **(A)** Representative photos of fluc-mcherry transduced KUGBM8-DMEM and KUGBM8-EF cell lines. **(B)** Plot demonstrating *in vitro* bioluminescence of KUGBM8-DMEM-FMC and KUGBM8-EF-FMC cell lines. **(C)** *In vivo* tumor growth curve of established cell lines. KUGBM8-EF and KUGBM8-DMEM cells were intracranially implanted into non-obese diabetic/severe combined immunodeficiency (NOD/SCID) mice and assessed for tumor growth for 75 days. Representative images of bilateral tumors of same mice from 4 to 75 days displaying normalized bioluminescent efficiencies acquired (blue to red indicates lower to higher radiance as photons/s/cm2/steradian). *N* = 4 per group. **(D)** Representative histological examination of tumors removed at the end of last imaging session, stained by H&E.

One important feature of cancer cell lines for studying tumorigenesis is their ability to form tumors *in vivo*. To test the tumor forming capacity of KUGBM8 cells, we labeled each cell line with Firefly Luciferase (Fluc) and mCherry encoding vectors and generated stable KUGBM8-DMEM and KUGBM8-EF cells by viral transduction (termed KUGBM8-DMEM-FmC and KUGBM8-EF-FmC, respectively). Both cell lines preserved their unique morphologies and preserved their viability upon transduction. While their mCherry expression was comparable as gauged by fluorescence microscopy (Figure 2A), their Fluc activities were different as measured by *in vitro* luminescence assays. The KUGBM8-DMEM-FmC cells displayed more Fluc activity/cell compared to KUGBM8-EF-FmC cells (Figure 2B).

For tumor implantation, each cell line was stereotaxically implanted into the same sites in the brain and tumor growth was monitored with non-invasive bioluminescence imaging over 75 days. While KUGBM8-DMEM-FmC cells did not grow to form any tumors, KUGBM8-EF-FmC cells were highly tumorigenic (Figure 2C). Histological examination of brain sections validated the presence of tumors derived KUGBM8-EF-FmC cells (Figure 2D). Hence, given their tumorigenic properties, we continued our experiments with KUGBM8-EF as a clinically relevant model.

### Patient Derived Primary Glioblastoma Cell Lines Respond to Temozolomide *in vitro and in vivo*

**FIGURE 3:**
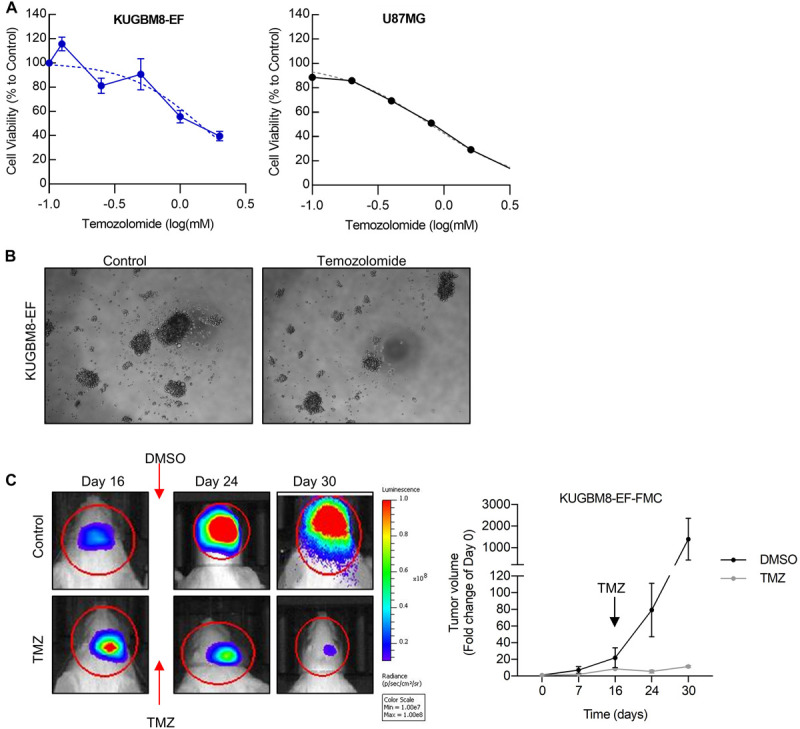
Temozolomide response of KUGBM8 cell line. **(A)** Dose response curve of KUGBM8-EF and U87MG to Temozolomide treatment for 5 days. IC_50_ was calculated by Graphpad Prism with non-linear regression analysis. **(B)** Representative photos of KUGBM8-EF after 4 days of treatment with 250 μM Temozolomide. **(C)** The efficacy of Temozolomide (TMZ) was tested *in vivo* in KUGBM8-EF implanted NOD/SCID mice. Cells were treated with pulsed TMZ (2 mg/kg) for 5 days and growth rates were detected by non-invasive bioluminescence imaging for 30 days. *N* = 3 mice per group. Statistical analysis between DMSO and TMZ treated mice were done by ANOVA, *p* < 0.05.

As frontline therapeutic for glioblastoma is TMZ, we first examined whether our new primary cell line is affected by TMZ treatment. TMZ response varies greatly for cell lines in the literature depending on the cell viability assay used (Lee, 2016). Temozolomide *in vitro* (125–2,000 μM) for 5 days and cell viability was detected using an ATP based reagent. 50% cell inhibition dosage (IC_50_) was found as 1.38 mM (Figures 3A,B) for KUGBM8-EF cells. With similar conditions IC_50_ for U87MG was found to be 787 μM (Figure 3A).

Next, we sought to study the dynamics of TMZ response of KUGBM8-EF *in vivo*. After establishing tumors *in vivo*, we treated mice with 2 mg/kg of TMZ for 5 days following the treatment regimen previously performed for U87MG model (Qi et al., 2013; Woo et al., 2014). In line with our *in vitro* results, we observed significant attenuation of tumor growth in TMZ-treated mice compared to DMSO-treated control group (Figure 3C). Taken together, these findings suggest that we generated a new patient derived primary glioblastoma model that is suited for studying therapy response *in vitro* and *in vivo*.

### Screen Among 1,200 FDA-Approved Drugs Reveals Topotecan as a Lead Drug for Primary Glioblastoma Cell Line

To find new drugs that can be incorporated into glioblastoma treatment, we utilized our newly generated KUGBM8-EF cell line and a chemical library of 1,200 FDA and EMEA approved drugs. The library is composed of drugs from 15 different therapeutic classes, namely, endocrinology, cardiovascular, immunology, diagnostic, metabolism, allergology, dermatology, gastroenterology, hematology, ophthalmology, neuromuscular, infectiology, respiratory, central nervous system, and oncology.

**FIGURE 4:**
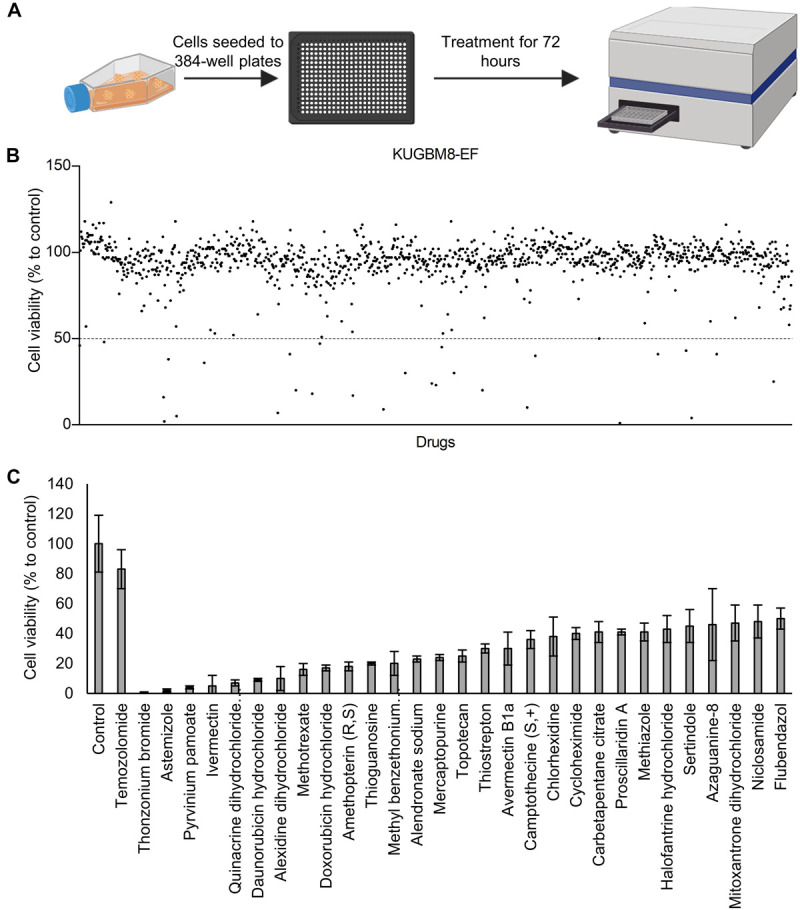
Drug repurposing screen performed on KUGBM8-EF cells**. (A)** Schematics of the screen. **(B)** Cell viability results detected by Cell Titer Glo (CTG) after 72 h of treatment. Drug treated cells were normalized to untreated wells. *N* = 4 for each condition. **(C)** Hits determined by 50% cell viability threshold. Error bars represent Normalized Standard Deviation. All hits have a significant *p*-value (*p* < 0.05) compared to control group, *t*-test.

Previous work, including ours, used this FDA-approved drug library in a variety of cancer screens in concentrations of 2–10 μM (Yip et al., 2006; Finlay et al., 2010; Senbabaoglu et al., 2016); accordingly, we chose 5 μM as optimal for this screen. KUGBM8-EF cells were treated with the library and viability was detected at 3 days (Figure 4A). Percent viability was determined by normalization to untreated controls, and a cut-off value of 50% was applied to select for the “hit” drugs (Figure 4B). Temozolomide at 250 μM dosage was included in the screen as a treatment control. 29 out of 1,200 drugs reduced KUGBM8-EF cell viability below 50% when normalized to control groups. These drugs were Thonzonium bromide (1 ± 0%), Astemizole (2 ± 1%), Pyrinium pamoate (4 ± 1%), Ivermectin (5 ± 7%), Quinacrine dihydrochloride dihydrate (7 ± 2%), Daunorubicin hydrochloride (9 ± 1%), Alexidine dihydrochloride (10 ± 8%), Methotrexate (16 ± 4%), Doxorubicin hydrochloride (17 ± 2%), Amethopterin (18 ± 3%), Thioguanosine (20 ± 1%), Methyl benzethonium chloride (20 ± 8%), Alendronate sodium (23 ± 2%), Mercaptopurine (24 ± 2%), Topotecan (25 ± 4%), Thiostrepton (30 ± 3%), Avermectin (30 ± 11%), Camptothecine (S, +) (36 ± 6%), Chlorhexidine (38 ± 13%), Cycloheximide (40 ± 4%), Carbetapentane citrate (41 ± 7%), Proscillaridin A (41 ± 2%), Methiazole (41 ± 6%), Halofantrine hydrochloride (43 ± 9%), Sertindole (45 ± 11%), Azaguanine-8 (46 ± 24%), Mitoxantrone dihydrochloride (47 ± 12%), Niclosamide (48 ± 11%), and Flubendazol (50 ± 7%) (Figure 4C and Table 3). When we categorized the hits into groups for their therapeutic effects, we observed that the major hit drugs belonged to infectiology and oncology (Table 3).

**TABLE 3:** Table of hits and their therapeutic classes and effects.

**Chemical name**	**%Cell viability** ± **%SD**	**Therapeutic class**	**Therapeutic effect**
Alexidine dihydrochloride	10 ± 8	Infectiology	Antibacterial
Methyl benzethonium chloride	20 ± 8	Infectiology	Antibacterial
Thiostrepton	30 ± 3	Infectiology	Antibacterial
Cycloheximide	40 ± 4	Infectiology	Antibacterial
Daunorubicin hydrochloride	9 ± 1	Infectiology, oncology	Antibacterial, antineoplastic
Doxorubicin hydrochloride	17 ± 2	Infectiology, oncology	Antibacterial, antineoplastic
Chlorhexidine	38 ± 13	Infectiology	Antibacterial, antiseptic
Ivermectin	5 ± 7	Infectiology	Antihelmintic
Avermectin B1	30 ± 11	Infectiology	Antihelmintic
Methiazole	41 ± 6	Infectiology	Antihelmintic
Niclosamide	48 ± 11	Infectiology	Antihelmintic
Quinacrine dihydrochloride	7 ± 2	Infectiology, metabolism	Antihelmintic, Antiparasitic
Astemizole	2 ± 1	Allergology	Antihistaminic
Halofantrine hydrochloride	43 ± 9	Metabolism	Antimalarial
Amethopterin (R,S)	18 ± 3	Immunology, oncology	Antineoplastic
Methotrexate	16 ± 4	Oncology	Antineoplastic
Thioguanosine	20 ± 1	Metabolism	Antineoplastic
Topotecan	25 ± 2	Oncology	Antineoplastic
Camptothecine (S,+)	36 ± 6	Oncology	Antineoplastic
Azaguanine-8	46 ± 24	Oncology	Antineoplastic
Mitoxantrone dihydrochloride	47 ± 12	Oncology	Antineoplastic
Alendronate sodium	23 ± 2	Metabolism	Antiosteoporetic
Sertindole	45 ± 11	Central Nervous System	Antipsychotic
Thonzonium bromide	1 ± 0	Dermatology	Antiseptic
Carbetapentane citrate	41 ± 7	Central nervous system	Antispastic, local anesthetic
Mercaptopurine	24 ± 2	Immunology, oncology	Immunosuppressant
Pyrvinium pamoate	4 ± 1	Metabolism	
Proscillaridin A	41 ± 2	Cardiovascular	
Flubendazol	50 ± 7	Metabolism	

**FIGURE 5:**
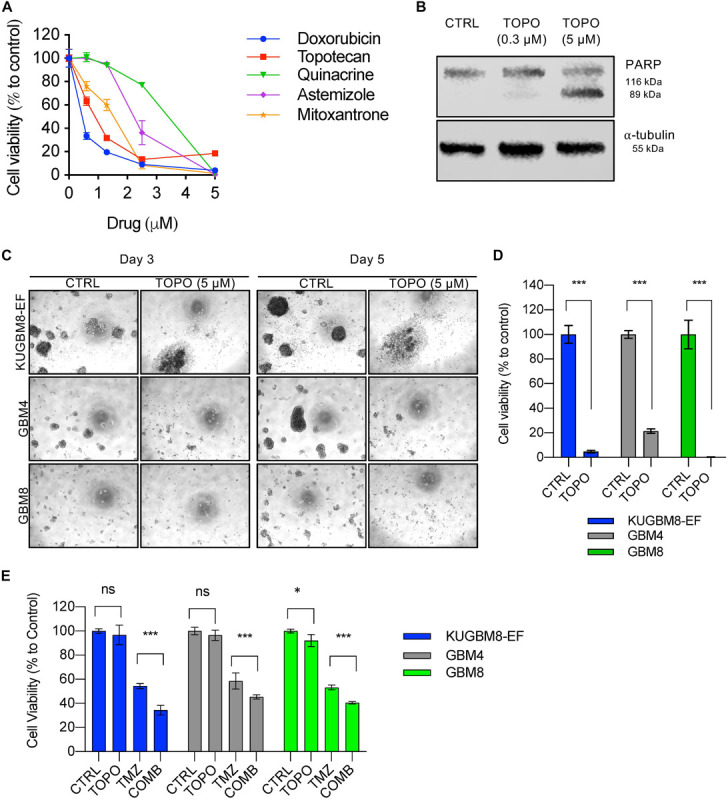
Hit validation revealed Topotecan as a potential apoptosis inducer in GBM **(A)** Dose response curves of selected hits treated for 3 days in 96-well format. **(B)** Detection of PARP cleavage after TOPO (0.3 or 5 μM) treatment for 24 h. α-tubulin was used as a loading control. **(C)** Representative photos of Topotecan (5 μM) response of 3 different primary GBM cell lines (KUGBM8-EF, GBM4, and GBM8) after 3 or 5 days of treatment. **(D)** Cell viability graphs of Topotecan (5 μM) treatment for 5 days on KUGBM8-EF, GBM4, and GBM8 cells. **(E)** The effect of low dose Topotecan (0.3 μM) combination with TMZ (250 μM for KUGBM8-EF, 65 μM for GBM4, and 10 μM for GBM8) on different primary GBM cell lines. Cell viability experiments were detected by CTG and viability was normalized to untreated controls. Error bars represent normalized standard deviation. n.s. stands for *p* > 0.05, ^∗^ stands for *p* < 0.05, ^∗∗∗^ stands for *p* < 0.0001, *t*-test.

Among the 26 hits, we chose Astemizole, Quinacrine, Doxorubicin, Mitoxantrone, and Topotecan for further studies. KUGBM8-EF cells responded to these drugs in a dose-dependent manner (Figure 5A). We then chose to continue with Topotecan, a topoisomerase 1 inhibitor, which has been shown as a radiation sensitizer in glioma cells (Pinel et al., 2006; Lesimple et al., 2009; Kortüm et al., 2016) and has blood brain barrier permeability (Baker et al., 1995; Stewart et al., 2004). Next, to investigate the mode of cell death in KUGBM8-EF cells, we checked cleaved PARP protein expression as an indicator of apoptosis. Accordingly, when KUGBM8-EF cells were treated with low doses of Topotecan (0.3 μM), cleaved PARP levels were low, however, with high dose Topotecan treatment (5 μM), cleaved PARP was markedly observed (Figure 5B). These results suggested that Topotecan induces apoptosis in KUGBM8-EF cells.

To investigate whether Topotecan can induce cell death in other primary Glioblastoma cell lines to similar degrees, we treated two different primary cell lines, GBM4 and GBM8, alongside KUGBM8-EF. We observed cell death and dissociation of spheres starting from day 3 of treatment in all cell lines (Figure 5C). When we measured cell viability after 5 days upon Topotecan treatment, we observed that cell viability was decreased below 25% for all three cell lines (Figure 5D). These results indicate that, our new cell line model, KUGBM8-EF, acts similarly to other known primary cells (Wakimoto et al., 2009).

Lastly, we investigated whether Topotecan can cooperate with TMZ and confers any additive effects on these primary cell lines. When we combined TMZ (250 μM for KUGBM8-EF, 65 μM for GBM4, and 10 μM for GBM8) with low dose Topotecan (0.3 μM) we observed significant cooperation between TMZ and Topotecan in all cell lines (Figure 5E). Taken together, Topotecan can be a good candidate to incorporate in glioblastoma treatment in the future.

## Discussion

For high throughput drug screening *in vitro*, feasible and cost-effective *in vitro* models are required. Established cell lines utilized for many years in cancer research have traditionally been cultured in the presence of serum. However, recent studies have shown that serum-based 2D cancer cell line models have different drug response patterns when compared to 3D cell line models (Horning et al., 2008; Hongisto et al., 2013). Especially for glioblastoma, serum based cell line culturing has been shown to cause depletion of cancer stem cell like cells (Singh et al., 2003; Seidel et al., 2014) and poor tumor growth abilities in mice (Lee et al., 2006; Xie et al., 2015). In this study, we established a patient-derived glioblastoma cell line, KUGBM8-EF, which was grown as spheres in neurobasal medium with supplements. Further analysis has shown that stem cell markers such as *SOX2, NESTIN*, and *PAX6* were highly expressed in KUGBM8-EF cells. In addition, KUGBM8-EF had high capacity for tumor growth in mice brain. Thus, we presented that this non-serum-based 3D cultures of KUGBM8-EF cells can be a useful option for drug screening. Since a repurposed drug comes with systemic toxicity knowledge, cytotoxicity problems can be circumvented when a drug is used for a novel indication. Therefore, we aimed to investigate potential glioblastoma therapy drugs out of a 1,200 FDA-approved drug library utilized at 5 μM dosage for 3 days. We identified 26 drugs that can reduce cell viability below the 50% threshold. Some of them were previously suggested as therapeutic candidates for glioblastoma such as Astemizole (Lee et al., 2016; Sales et al., 2016), Pyrivinium pamoate (Hothi et al., 2012; Lamb et al., 2015; Venugopal et al., 2015), Ivermectin (Liu et al., 2016), Doxorubicin hydrochloride (Jiang et al., 2014), Campthothecine (Lee et al., 2013), Niclosamide (Wieland et al., 2013), Proscillaridin A (Denicolaï et al., 2014), and Topotecan (Patel et al., 2013; Bernstock et al., 2017). Unfortunately, TMZ was not able to decrease cell viability less than 50%, even with the 250 μM dosage added manually to the screen, attesting to the important need for identifying novel therapy options. Topotecan is a topoisomerase I inhibitor derived from Campthothecin, and is approved by FDA for cancer treatments such as cervical, ovarian and small cell lung cancer (Pommier, 2006; Paton et al., 2010). Topotecan is also a blood brain barrier permeable agent (Baker et al., 1995; Stewart et al., 2004), which is a major concern for treatment options for brain cancers. Indeed, there is considerable clinical interest in using Topotecan as it is being tested in phase II clinical trials in combination to TMZ and/or other agents in recurrent gliomas (NCT00002814, 2011; NCT00541138, 2011; NCT00610571, 2012; NCT01931098, 2020). In accordance with its well-established effects in the literature, we demonstrated that Topotecan can induce cell death in various primary glioblastoma cell lines including our new one, KUGBM8-EF. In addition, Topotecan can be combined with TMZ in low dosages to reduce cell viability *in vitro*.

Together, our study adds a new primary glioblastoma cell line model to the growing list of primary lines. Our identification of Topotecan as a lead drug in this model suggests the utility of KUGBM8-EF cells as tool to discover novel glioblastoma therapeutics in the future.

## Conclusion

In this study we established new primary glioblastoma cell lines, KUGBM8-DMEM and KUGBM8-EF, utilizing different medium conditions. Here, we showed that culturing freshly derived tumor tissue in serum-free neurogenic medium provides a better alternative to culturing these cells with serum. We also showed that KUGBM8-EF cells can readily grow *in vivo* and respond to frontline TMZ chemotherapy, suggesting its future applications to study the biology of glioblastoma. Performing a high throughput screen with non-serum based KUGBM8-EF with clinically approved drugs, we identified 26 lead drugs that can significantly reduce cell viability Among these, Topotecan, a topoisomerase inhibitor with blood brain barrier permeability, revealed the most efficacy as a single agent or in combination with TMZ.

## Data Availability Statement

The raw data supporting the conclusions of this article will be made available by the authors, without undue reservation.

## Ethics Statement

The studies involving human participants were reviewed and approved by the Koç University Institutional Review Board. The patients/participants provided their written informed consent to participate in this study. The animal study was reviewed and approved by the Koç University Institutional Review Board.

## Author Contributions

FS and TB-O conceived and planned the experiments, and took lead in writing the manuscript. FS, ACA, AC, FS-P, EB-Y, and İS carried out the experiments and data interpretation. All authors provided critical feedback and helped shape the research, analysis, and manuscript.

## Conflict of Interest

The authors declare that the research was conducted in the absence of any commercial or financial relationships that could be construed as a potential conflict of interest.
