# Supplementary material for: Drug Repositioning Screen on a New Primary Cell Line Identifies Potent Therapeutics for Glioblastoma
Source: Front Neurosci. 2020 Dec 17;14:578316. doi: 10.3389/fnins.2020.578316 (PMC7773901; doi:10.3389/fnins.2020.578316)
Supplement: Supplementary file 1 [file Table_4.DOCX]

| **Gene** | **Primer Sequence** | |
| --- | --- | --- |
| TERT | Forward | GGCACGGCTTTTGTTCAGAT |
|  | Reverse | ACATGCGTGAAACCTGTACG |
| GFAP | Forward | GGACGCCATTGCCTCATACT |
|  | Reverse | TGCCTATAGACAGGAAGCAGA |
| p53 | Forward | GGTGACACGCTTCCCTGGATT |
|  | Reverse | AGGGGGACAGAACGTTGTTTTCAG |
| PTEN | Forward | GGCGGAACTTGCAATCCTCAG |
|  | Reverse | CACCACACACAGGTAACGGCT |
| PDGFRA | Forward | GCTCAGCCCTGTGAGAAGAC |
|  | Reverse | ATTGCGGAATAACATCGGAG |
| Sox2 | Forward | CCCAGCAGACTTCACATGT |
|  | Reverse | CCTCCCATTTCCCTCGTTTT |
| NESTIN | Forward | GCGTTGGAACAGAGGTTGGA |
|  | Reverse | TGGGAGCAAAGATCCAAGAC |
| GAPDH | Forward | AGCCACATCGCTCAGACAC |
|  | Reverse | GCCCAATACGACCAAATCC |
| Pax6 | Forward | ATCCGAGATTTCAGAGCCCCA |
|  | Reverse | CGCCCGTTGACAAAGACACCA |

**Supp. Table 1.** List of qRT-PCR primers
